# From Evolution to Pathogenesis: The Link Between β-Barrel Assembly Machineries in the Outer Membrane of Mitochondria and Gram-Negative Bacteria

**DOI:** 10.3390/ijms13078038

**Published:** 2012-06-28

**Authors:** Jhih-Hang Jiang, Janette Tong, Kher Shing Tan, Kipros Gabriel

**Affiliations:** Department of Biochemistry and Molecular Biology, Monash University, Clayton Campus, Melbourne 3800, Australia; E-Mails: jhih-hang.jiang@monash.edu (J.-H.J.); janette.tong@monash.edu (J.T.); kher.tan@monash.edu (K.S.T.)

**Keywords:** β-barrel proteins, mitochondria, bacteria, outer membrane, protein transport, protein folding, evolution

## Abstract

β-barrel proteins are the highly abundant in the outer membranes of Gram-negative bacteria and the mitochondria in eukaryotes. The assembly of β-barrels is mediated by two evolutionary conserved machineries; the β-barrel Assembly Machinery (BAM) in Gram-negative bacteria; and the Sorting and Assembly Machinery (SAM) in mitochondria. Although the BAM and SAM have functionally conserved roles in the membrane integration and folding of β-barrel proteins, apart from the central BamA and Sam50 proteins, the remaining components of each of the complexes have diverged remarkably. For example all of the accessory components of the BAM complex characterized to date are located in the bacterial periplasm, on the same side as the *N*-terminal domain of BamA. This is the same side of the membrane as the substrates that are delivered to the BAM. On the other hand, all of the accessory components of the SAM complex are located on the cytosolic side of the membrane, the opposite side of the membrane to the *N*-terminus of Sam50 and the substrate receiving side of the membrane. Despite the accessory subunits being located on opposite sides of the membrane in each system, it is clear that each system is functionally equivalent with bacterial proteins having the ability to use the eukaryotic SAM and *vice versa*. In this review, we summarize the similarities and differences between the BAM and SAM complexes, highlighting the possible selecting pressures on bacteria and eukaryotes during evolution. It is also now emerging that bacterial pathogens utilize the SAM to target toxins and effector proteins to host mitochondria and this will also be discussed from an evolutionary perspective.

## 1. Basic Features of the Bacterial and Mitochondrial Membranes

Gram-negative bacteria contain two lipid bilayers which envelope the periplasm: the inner membrane (IM) and outer membrane (OM). The asymmetric outer membrane is composed of lipopolysaccharides (LPS) in the outer leaflet and phospholipid in the inner leaflet while the symmetric inner membrane is composed of a phospholipid bilayer. The outer membrane predominantly harbors β-barrel fold proteins whereas α-helical based transmembrane proteins are primarily found in the inner membrane. The outer membrane provides the physical barrier from the outside world, but also must maintain selective permeability for the uptake of nutrients to support physiological functions, for the release of virulence factors and in some cases for multidrug resistance. This is achieved by the outer membrane proteins [1,2]. Evolutionarily, mitochondria are derived from an ancient α-proteobacterium through an endosymbiotic relationship that was initiated billions of years ago [3–5]. They have retained their double membrane structure that forms the inner and outer membranes, which enclose two aqueous environments, the intermembrane space and the matrix. This allows for the separation of biochemical processes and hence mitochondria perform many roles in eukaryotic cells. To carry out these functions, nutrients, biochemical intermediates, lipids and proteins must traverse the mitochondrial membranes. Outer membrane proteins (OMPs), particularly β-barrel proteins, play a key role in these transport processes [6].

## 2. β-Barrel Proteins and the Assembly in the Bacterial and Mitochondrial Outer Membrane

β-barrel proteins are defined as anti-parallel β-strands forming larger β-sheets that are connected through hydrogen bonds between the transmembrane strands that confer extreme stability [7]. As most β-barrels are membrane spanning proteins, it is typical for them to possess amino acids with hydrophobic side chains on their surface that abuts the phospholipids, while hydrophilic or charged residues often face the central cavity [1,7,8].

The assembly of β-barrel OMPs is mediated by sophisticated molecular machineries. In Gram-negative bacteria, nascent polypeptides are synthesized with an amino-terminal signal peptide by cytoplasmic ribosomes. These precursors are bound by factors such as SecB and SecA or the chaperone DnaK and transported to the secretion machinery (Sec). The Sec translocates proteins into the periplasm. Once in the periplasm, the signal peptide is processed, and precursor proteins associate with the chaperones SurA and Skp which not only transfer proteins to the BAM complex for outer membrane insertion but also prevent their aggregation [9]. A recent study has also shown that chaperones also play an important role in assisting folding and assembly of β-barrel precursor proteins, using *in vitro* reconstitution techniques with BAM and its substrates [10].

Similarly, in mitochondria, precursor β-barrel proteins are also synthesized by cytosolic ribosomes and are bound by chaperones in the cytosol [11]. These precursor substrates are transported to the receptor subunits of the Translocase of the Outer Mitochondrial membrane (TOM). The TOM complex translocates substrate proteins across the outer membrane into the intermembrane space. The small chaperones Tim9/10 or Tim8/13 associate with substrates, in a manner which is functionally analogous to that performed by SurA and Skp in bacteria, for the transfer to the Sorting and Assembly Machinery (SAM) complex at the mitochondrial outer membrane [12–17].

## 3. The β-Barrel Assembly Machinery in Gram-Negative Bacteria

In bacteria, the Omp85 family of protein assembly machines is the key and essential component of outer membrane β-barrel protein insertion processes. Omp85 protein homologues are found in bacteria and eukaryotes, including fungi, plants (mitochondria and chloroplasts) and animals [15–19]. Study of the BamA protein in *N. meningitidis* [18] and *Escherichia coli* [20–22] first revealed the essential role of Omp85 protein machines in folding and assembly of outer membrane proteins. BamA is highly conserved throughout all Gram-negative bacterial species [18]. Structural predictions relating to BamA suggest that it is composed of two parts: a large soluble *N*-terminal domain protruding to the periplasm; and the carboxy-terminal β-barrel domain sitting in the OM. The large soluble domain has five repeat POTRA (polypeptide transport-associated) domain, these repeat domains are suggested to bind unfolded β-barrel proteins [23]. The deletion of each POTRA domain exerts different effects to complex stability and substrate binding. POTRA domains 2 to 4 are important for the interaction with the partner protein in the BAM complex, BamB, whereas deletion of POTRA 5 specifically affects association with other lipoprotein partners [24,25].

Defects in BAM complex function have many deleterious primary and secondary effects, including accumulation of unfolded protein substrates and reduced LPS and phospholipid incorporation into the outer membrane. Failures in these processes would cause accumulation of these factors at the inner membrane [26]. Recent evidence indicates BamA acts as the assembly hub for the formation of an LptD/E complex in the outer membrane, which is subsequently essential for LPS biogenesis [27]. In *E. coli*, BamA (previously referred as YaeT) is associated with four lipoproteins BamB, C, D and E (previously known as YfgL, NlpB, YfiO and SmpA respectively) [20,28–30]. The BAM is also involved in autotransporter protein biogenesis, which is not surprising as autotransporters have a *C*-terminal β-barrel domain [31,32]. The *N*-terminus of autotransporter proteins consist of passenger domains which are translocated through the folded barrel formed by the *C*-terminus and usually act as virulence factors during infection. The BAM was shown to be associated with stalled autotransporter substrates created by an *in vivo* site-specific photocrosslinking approach. It is hypothesized that the β-barrel domain initially interacts with chaperones in the periplasm and then passed to BamA, BamB and BamD sequentially. As both BamB and BamD are still found crosslinked to substrates at later time points during chase experiments, it has been suggested that they are involved in the late stages of folding/assembly. The passenger domain of autotransporter EspP can transiently interact with the channel BamA and the release of passenger domain seems to be a checkpoint for the completion of assembly of β-barrel domain [31,32].

## 4. The Sorting and Assembly Machinery (SAM) in Mitochondria

The key molecule of the eukaryotic SAM is the β-barrel core protein, Sam50, the homolog of the bacterial protein BamA. Sam50 is predicted to have one POTRA domain that faces the mitochondrial intermembrane space, the equivalent of the bacterial periplasm. Thus, assembly of β-barrels occurs from the same face of the membrane in bacteria and mitochondria as substrates are first imported by the TOM complex into the intermembrane space before they engage with the SAM. This suggests that the functional features of the eukaryotic system have been retained from the endosymbiont [33,34]. The POTRA domain of Sam50 was once reported as a signal receptor for β-barrel protein as deletion of a short segment caused a severe phenotype and a loss in SAM-substrate interaction [35]. The role of POTRA in Sam50 is now thought to facilitate the folded β-barrel release into the mitochondrial outer membrane instead of having a signal receptor role as deletion of the entire POTRA domain results in little change from the wild type phenotype and does not affect the kinetics of β-barrel assembly *in vitro* [36,37].

**Figure 1:**
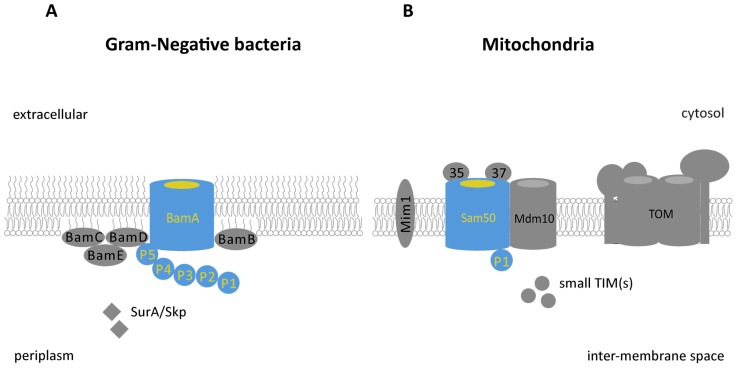
Comparison of the β-barrel protein assembly machineries in Gram-negative bacteria and eukaryotes (**A**) In Gram-negative bacteria, BamA is the core subunit of the BAM complex. BamA forms a complex with lipoproteins, BamB, C, D and E in *E. coli*. The BAM complex plays an important role in outer membrane biogenesis including the insertion of autotransporters; (**B**) In eukaryotes, the core channel of SAM, Sam50, is the homologue of BamA. In mitochondria, Sam50 forms a complex with the metaxins, Sam35 and Sam37. Mdm10 interacts with SAM to mediate the biogenesis of TOM. Mim1 transiently interacts with the SAM complex and is important for the biogenesis of α-helical transmembrane domain containing proteins. BamA has five repeat POTRA domains (P1–P5) while Sam50 only has one (P1). In eukaryotes the accessory subunits are located on the cytosolic side of the outer membrane whilst in bacteria BamB to E are located on the periplasmic face of the membrane. Color key-grey denotes components not conserved between prokaryotes and eukaryotes, blue denotes conserved proteins.

In addition to the central protein, Sam50, the SAM complex is also composed of two peripheral subunits that face the cytosol, Sam35 and Sam37. Two other integral membrane proteins also transiently act in the β-barrel folding pathway, Mdm10 and Mim1 [11,38,39]. Only Sam50 is evolutionarily conserved from Gram-negative bacteria to mitochondria (Figure 1) [15–17]. Moreover the peripheral subunits, Sam35 and Sam37, are actually located on the opposite side of the membrane to the intermembrane space and hence the POTRA domain. This is in contrast to the bacterial system where BamB to E have large domains in the periplasm. Sam35 is a receptor that binds the β-signal of the substrate β-barrel proteins, stabilizing the substrate proteins in the SAM complex whilst strands are being inserted into the membrane [37,40]. Sam37 promotes the release of β-barrel substrate proteins from SAM complex at a later stage of folding [40]. The SAM complex is not only considered to be a β-barrel protein folding station. It is also associated with the post-folding transfer and incorporation of proteins into their respective native complexes; it also plays a role in connections and communication with other organelles. Hence it is considered a “hub” for membrane protein biogenesis and organelle communication [41–43]. The metaxin1 and 2 proteins in mammalian cells are considered to perform similar roles to Sam35 and Sam37 [44].

Critically, the SAM complex serves as the initial platform for assembly of the TOM complex that is the entry portal for all imported mitochondrial proteins. The central component of the TOM complex, the protein conducting pore, Tom40, is also a β-barrel protein. Tom40 needs to be assembled with a series of receptor subunits including, Tom20, Tom22 and Tom70, as well as three small Tom proteins (Tom5, Tom6, and Tom7) that seem to have a complex organising role [38,45–48].

Two other proteins which form transient associations with the SAM are Mdm10 and Mdm12 [38,39,49]. Collectively these proteins are also important for the formation of the endoplasmic-reticulum mitochondria encounter structure (ERMES) [38,39,41,42,49–53]. Mim1 also interacts with the SAM complex and is important for the biogenesis of outer membrane tethered α-helical transmembrane domain proteins [54,55].

A recent report also implicates Sam50 in the maintenance of cristae structure and respiration by serving as a bridging point between the outer and inner mitochondrial membrane [56]. This emphasizes the multi-functional role of the SAM in mitochondrial biogenesis, function and maintenance. These multiple roles can be explained by the evolutionary view that eukaryotic cells developed new mitochondrial functions based on pre-existing protein molecules as scaffolds as well as keeping the original functions of these molecules. The role of Sam50 as a β-barrel protein insertion and folding machine in addition to the many other roles of the SAM complex are testament to this theory.

## 5. How Functionally Conserved Are the BAM and SAM?

Since the discovery of both BamA and Sam50, many bacterial β-barrel proteins have been used to test the ability of SAM complex to fold bacterial β-barrel proteins in mitochondria [57–60] (Table 1). The 8-stranded monomeric barrel OmpA, the 16-stranded OmpC and PhoE from of *E. coli*, and Omp85 from *Neisseria meningitidis* have been reported to target to mitochondria when they were expressed in the eukaryote *Saccharomyces cerevisiae*. Sam50 and Sam37 were shown to be required for PhoE assembly using genetically modified yeast strains [57]. The *C*-terminal residue of PhoE, which is required for the assembly in bacteria, is also suggested to be important for the trimeric formation of PhoE in mitochondria [57,61]. On the other hand, expression of OmpA, OmpC or PhoE in mammalian cell lines fails to result in successful targeting and assembly in mitochondria. The Omp85 and PorB proteins from *Neisseria gonorrhoeae* are recognized and inserted and folded into the mitochondrial outer membrane by the SAM in mammalian and yeast derived mitochondria [58,60]. Furthermore, expression of a different class of bacterial β-barrel, where more than one protein subunit is required for complete barrel formation, was also shown to be possible in mitochondria. The barrel domain of the YadA autotransporter from *Yersinia enterocolitica* expressed in the yeast *S. cerevisiae* showed that eukaryotes could assemble these fragments into a barrel. Trimeric YadA assembles as a 12-stranded β-barrel with a contribution of 4-strands from each YadA. The assembly process requires the intermembrane space chaperone proteins and the SAM complex [59]. The reasons why some and not other bacterial proteins can be imported and folded in eukaryotic mitochondria are not known but is likely to be related to the presence or absence of signal sequences that target them to mitochondria and secondly be able to be recognized by the SAM.

Reciprocal experiments examining the folding and assembly of eukaryotic β-barrel proteins in bacteria have also shown that bacterial machineries can assemble and fold eukaryotic β-barrel proteins in the bacterial outer membrane. The eukaryotic mitochondrial porin, from *Neurospora crassa*, one of the most abundant β-barrel outer mitochondrial membrane proteins of eukaryotes, can be assembled into the bacterial outer membrane [69].

## 6. β-Barrels—Evolution and Pathogenesis

Mitochondria play dominant roles in cell death and energy metabolism [70]. During bacterial infection, many effector proteins are transferred to host cells for the manipulation of cell function [71]. It is therefore not surprising that mitochondria are targets of bacterial toxins [72]. The fact that bacterial β-barrels can in fact be assembled in mitochondria is not only intriguing from an evolutionary perspective, but it is now becoming clearer. Evolutionary conserved principles are used by some bacteria to target β-barrel toxins to host mitochondrial outer membranes via the SAM.

The bacterial outer membrane protein PorB from *Neisseria gonorrhoeae* (gonococcal) and *Neisseria meningitidis* (meningococcal) have been found to be targeted to the mitochondria during infection [58,60,62–66]. PorB is a β-barrel protein composed of 16 β-strands and has similar electrophysiological activity as eukaryotic porins that can be regulated by purine triphosphate nucleotides [73,74]. The localization of PorB in mitochondria was once controversial. Meningococcal PorB was suggested to target to the mitochondrial outer membrane while gonococcal PorB was once reported to randomly insert into mitochondrial inner membrane [62,65]. The exact localization of PorB in mitochondria was recently examined using a novel method combining *in vitro* mitochondrial import assays and β-barrel mobility gel shift assays. PorB assembles in the mitochondrial outer membrane using the SAM core subunit Sam50 [60]. The molecular mechanisms behind PorB targeting to mitochondria can be attributed to the evolutionary link between mitochondria and their bacterial ancestors. PorB enters mitochondria through the TOM complex like all endogenous eukaryotic β-barrels [64]. After entry into mitochondria, the chaperone proteins in the intermembrane space, the small Tims, are required for transport of PorB to the SAM in the outer membrane [60]. Interestingly, the accessory subunits of the SAM, Sam35 and Sam37, which are important for eukaryotic β-barrel folding, are not required for PorB assembly in mitochondria [60]. Only the core SAM subunit, Sam50, is required. Furthermore, unlike eukaryotic β-barrels, PorB does not have a strict eukaryotic like β-signal at the carboxy-terminus [37,60]. This could be explained by the fact that β-signal regions that evolved in mitochondria and bacteria have diverged, with the signal in eukaryotes co-evolving with Sam35 and Sam37. The signal regions in eukaryotes could have a greater role in the regulation of the rate of folding but may not be absolutely required and hence are not found universally in bacteria. Although it is generally agreed that PorB can localize to the mitochondria, its exact function remains controversial with both pro and anti-apoptotic effects reported [62,66].

Another pathogenic β-barrel Omp38 (AbOmpA) from *Acinetobacter baumannii* has also been found to target mitochondria and reported to promote cell death [67]. AbOmpA is a bacterial outer membrane protein which has a *C*-terminal OmpA-like domain which can bind peptidoglycan [75]. The localization of AbOmpA in mitochondria and the molecular mechanisms behind import are still unclear but it would be anticipated that it may also use the SAM for its membrane folding/insertion.

In contrast to traditionally studied β-barrel proteins, other β-stranded bacterial proteins that are not likely to use the SAM also exist. Panton-Valentine leukocidin (PVL) from *Staphylococcus aureus* has also been identified as a mitochondrial targeted that induces apoptosis in neutrophils [68]. PVL is a bi-component toxin composed of two subunits, LukF-PV and LukS-PV, with the ability to form β-barrel in membranes [76,77]. However, ability of PVL to form pores at either the mitochondrial inner or outer membrane is yet to be demonstrated. Structural information also suggests that PVL is unlikely to utilize the SAM as it is predicted to be structurally and functionally analogous to other leukocidin and γ-haemolysin like toxins that firstly form multimers and a channel like structure at the membrane surface before they insert into the membrane without assistance from other machineries [78].

## 7. Concluding Remarks and Future Directions

Hosts and pathogens co-evolve, with advantageous traits of hosts or pathogens selected for in a given population. For bacterial pathogens this may mean that for example; a surface factor is altered to better evade host immune defences; or a modified and more potent toxin is produced. More potent toxins could be the result of a mutational change that alters the way a toxin interacts with its host target protein or pathway, but it may also be the result of changing the efficiency of transport of the toxin to its target site. We propose that some pathogens, through selective forces, have continued to produce β-barrel toxins that can be transported to mitochondria with high efficiency. As mitochondria have diverged from their bacterial ancestors and adapted their mechanism for the import/assembly of β-barrel outer membrane proteins to their cellular context, the bacterial pathogens that produce β-barrel toxins that are targeted to mitochondria have by necessity also changed. Any changes in the way the SAM recognizes β-barrel proteins, in particular if this results in changes in any signal regions of the β-barrel substrate proteins, need to be countered by the bacterial pathogens. It will be interesting to compare the efficiencies of import and assembly of β-barrel toxins into mammalian mitochondria with non-toxin bacterial β-barrels on a large scale to assess for the differences as our list of bacterial β-barrel toxins increases.

Research in the area of β-barrel protein biogenesis in Gram-negative bacteria and mitochondria during the past decade highlights the link between the two β-barrel assembly machineries, the BAM and SAM, respectively. The last ten years of research have revealed much about these remarkable machines, though future research will elucidate many of the mechanistic details that are still unclear. It is now emerging that due to their evolutionary heritage, mitochondria are targets of bacterial β-barrel toxins. As more β-barrel bacterial toxins are discovered, the battery of substrates to probe SAM function will increase, hopefully allowing us to understand how the exact mechanisms utilised by BAM and SAM to insert and fold proteins in the membrane in addition to better understanding mechanisms at play during infection.

## Figures and Tables

**Table 1:** Tests of bacterial β-barrel assembly in mitochondria.

Bacterial species	β-barrel protein	References
Bacterial βbarrels target to mitochondria during infection		
*Neisseria gonorrhoeae*	PorB	[58,60,62–64]
*Neisseria meningitidis*	PorB	[60,65,66]
*Acinetobacter baumannii*	Omp38 (AbOmpA)	[67]
*Staphylococcus aureus*	Panton-Valentine leukocidin (PVL)	[68]
Bacterial β-barrels tested to be expressed in mitochondria		
*E. coli*	PhoE, OmpA, OmpC	[57,58]
*Yersinia enterocolitica*	YadA	[59]
*Neisseria meningitidis*	Omp85(BamA)	[57]
*Neisseria gonorrhoeae*	Omp85(BamA)	[58]
